# Towards a Socialization of the EU's New Economic Governance Regime? EU Labour Policy Interventions in Germany, Ireland, Italy and Romania (2009–2019)

**DOI:** 10.1111/bjir.12522

**Published:** 2020-02-18

**Authors:** Jamie Jordan, Vincenzo Maccarrone, Roland Erne

**Affiliations:** ^1^ Jamie Jordan is at De Montfort University Leicester; ^2^ Vincenzo Maccarrone and Roland Erne are at the School of Business and Geary Institute for Public Policy University College Dublin

## Abstract

In response to the last recession, the European Union (EU) adopted a new economic governance (NEG) regime. An influential stream of EU social policy literature argues that there has been more emphasis on social objectives in the NEG regime in more recent years. This article shows that this is not the case. It does so through an in‐depth analysis of NEG prescriptions on wage, employment protection and collective bargaining policy in Germany, Italy, Ireland and Romania between 2009 and 2019. Our main conclusion is that the EU's interventions in these three industrial relations policy areas continue to be dominated by a liberalization agenda that is commodifying labour, albeit to a different degree across the uneven but nonetheless integrated European political economy. This finding is important, as countervailing transnational trade union action is the more likely, the more there is a common threat. Even so, our contextualized analysis also enables us to detect contradictions that could provide European labour movements opportunities to pursue countervailing action.

## 1 Introduction

While European industrial relations may be a multilevel configuration, most industrial relations scholars agreed until recently that it ‘is evidently not a vertically integrated system’ (Leisink and Hyman 2005: 281; Marginson and Sisson 2004). On the eve of the financial crisis, even European social partners perceived ‘the sole fact that public authorities and academics showed interest in industrial relations under the banner of governance… as too much intervention’ (Léonard *et al*. 2007: 7). However, only three years later, the response of the European Union (EU) to the crisis proved wrong those who argued that gridlocks ingrained into the EU Treaties would prevent ‘the reconstruction of a system of economic regulation at the level of the larger unit’ (Scharpf 1999: 45). When political leaders realized that the ‘invisible hand’ behind market integration created threatening imbalances rather than economic convergence, their take on EU governance shifted dramatically. Thus, the single‐market regime, which shaped the political strategy for uniting Europe since 1985 (Jabko 2006), was complemented by a new economic ‘governance’ regime (Erne 2015; Jabko 2019).

The crisis led to an emergency in which ‘the impending catastrophe empowers and even forces the Europe builders to exploit legal loopholes to open the door to changes’ (Beck 2013: 26–27). As a result, the institutional gridlocks described by Scharpf as preventing further political integration were shaken off (Joerges 2013). First, the European Commission approved massive bank bailouts, which were at odds with ‘ordoliberal’ EU Treaty provisions that were arguably meant to prevent state aid for private corporations as well as excessive budget deficits. Subsequently, the Commission, European Parliament and Council used a latent (Maastricht) Treaty clause — ‘The European Parliament and the Council, acting by means of regulations in accordance with the ordinary legislative procedure, may adopt detailed rules for the *multilateral surveillance’* (TFEU, Art. 121 [6], emphasis added) — in order to set up a new, much more ‘vertical’ economic governance regime (Erne 2018: 237). This led to the adoption of the ‘Six Pack’ of EU laws by the European Parliament and Council, which gave the Commission wide‐ranging policy intervention and sanctioning powers, not only in order to counter ‘excessive’ budget deficits of EU member‐states, but also to ensure the ‘*proper functioning* of economic and monetary union’ (Regulation No 1176/2011, Art. 2, emphasis added).

While earlier EU directives in the area of social and labour policy left member‐states considerable scope for interpretation and adaptation, EU policy prescriptions based on the Six‐Pack (2011), the Two‐Pack (2013) and subsequent regulations on economic governance have left member‐states with ‘excessive deficits’ or ‘excessive macroeconomic imbalances’ much less room for manoeuvre. As the really existing European market failed to bring about the desired convergence of economic policies, the new EU economic governance regime aims to implement ‘proper’ economic governance by fiat (Erne 2015). Tellingly, in 2012, an official from DG Economics and Finance stated that the Commission lost its faith in self‐governing markets, which would explain why the Commission had to intervene politically.^1^

Since 2010, the economic governance of the EU has thus undergone what the former Commission President, José Manuel Barroso, labelled a ‘silent revolution’ (ANSA 2010). Specifically, he was referring to the new Six Pack of EU laws mentioned previously, which strengthen the enforcement procedures of the Stability and Growth Pact (SGP) to control member‐states’ public finances and introduced a new Macroeconomic Imbalance Procedure (MIP) attempting to prevent and correct macroeconomic imbalances. These measures came on top of the Europe 2020 strategy — replacing the Lisbon Agenda in 2010 — with its agenda of pursuing ‘smart, sustainable and inclusive growth’ through a European coordination of national social and economic policies. As all three faces of this new regime are policy interdependent, in 2011, the EU introduced the European Semester (the Semester, hereafter), a yearly cycle of country‐specific recommendations (CSRs), surveillance and enforcement that integrates all EU interventions relating to the SGP, the MIP and the Europe 2020 strategy in one document.

There is now a growing body of literature that traces the evolving policy content of the Semester, especially the Council Recommendations on National Reform Programmes (CSRs), which the Commission proposes in May and the Council adopts in July every year. Cohering around what we label ‘the socialization debate’, several social policy scholars detect a partial but persistent ‘socialization’ of the Semester, especially since a more ‘political’ Commission (Juncker 2014) took over from the previous administration headed by Barroso. But while Jean Claude Juncker's candidacy speech included strong statements that aimed at countering the growing popular discontent caused by the EU's emergency interventions,^2^ our analysis of the EU's new economic interventions in three main industrial relations areas leads us to conclusions that contradict the ‘socialization’ thesis that claims that there have been more and more CSRs with an increasing emphasis on social objectives across all employment and social policy areas (Zeitlin and Vanhercke 2018).

Our contrasting findings result from a different research design that addresses serious limitations present within the analysis of the socialization literature. These limitations are: (i) a problematic conceptual reflection on what the Semester actually is; (ii) a failure to take account of the varied legal status of CSRs; (iii) a lack of analysis that situates the CSRs in the context of the receiving country's position in the EU's uneven but nonetheless integrated political economy. Our aim is to address these issues, making important conceptual, methodological and empirical contributions. In doing so, our main conclusion is that the EU's substantive policy interventions in the area of industrial relations and labour market regulation continue to be dominated by a liberalization agenda, which is ultimately leading to the further commodification of labour. This finding is not just of academic interest, as the apparent socialization of the Semester arguably led the European Trade Union Confederation (ETUC) to abandon its initial adverse stance towards it (Erne 2015). At its 2019 Congress, the ETUC not only acknowledged that ‘a process of making the European Semester more social has started’ (ETUC 2019: 23; see also Golden 2019). It also committed its affiliates to participate in it ‘in a spirit of dialogue and solidarity’ (ETUC 2019: 24). The ‘socialization’ thesis must therefore be taken seriously, also due to its relevance in the EU policy debate.^3^ Even so, our contextualized analysis also enables us to detect contradictions that could provide European labour movements with opportunities to pursue countervailing action, which we discuss at the end of the article.

To substantiate our main argument, the structure of the article is as follows: the next section will discuss the ‘socialization debate’ literature. The second section will outline and justify our alternative conceptual and methodological framework. The third section will apply our framework and provide a primary analysis of EU‐level documentation for the countries and policy areas mentioned. The fourth section summarizes the arguments of the article, before finally discussing the implications of our findings for labour movements and the European integration process.

## 2 The socialization debate and its analytical limits

Since the introduction of the Semester in 2010, there has been a growing body of EU studies literature that is concerned with analysing the processes and outcomes of policy formation and recommendation. This literature relates especially to so‐called CSRs which are drafted by the Commission and adopted by the Council (of finance ministers) every July. The dominant thread of this literature is focused on analysing whether this yearly cycle has been ‘socialized’ (Bekker 2015; Zeitlin and Vanhercke 2014, 2018). The main proponents of this literature argued that since 2013 we have unequivocally witnessed a partial but progressive socialization of the Semester, both in terms of its substantive content and its governance procedures (Zeitlin and Vanhercke 2018). Socialization in the terms of these authors means a significant growth of ‘social objectives in the Semester's policy orientations and messages’ (Zeitlin and Vanhercke 2018: 149).

This central ‘socialization’ argument has been challenged by others who are less optimistic about the social content of EU economic governance policy prescriptions, especially CSRs (Copeland and Daly 2018; Crespy and Vanheuverzwijn 2017; Dawson 2018; Erne 2018, 2015). For instance, even when excluding CSRs predominantly focusing on budgeting and fiscal governance, Copeland and Daly conclude ‘EU social policy as enunciated through the CSRs is much more oriented to supporting market development than it is to correcting for market failures’ (2018: 2). Considering their exclusions meant that there were only 290 of the 656 CSRs issued left to analyse, they are right to recognize that ‘the scale of the exclusions is itself indicative of the focus of the CSRs on areas of policy that are not social’ (Copeland and Daly 2018: 5). Therefore, any interpreted socialization of the Semester should not exclude fiscal or macroeconomic CSRs *a priori*. For this reason, we have chosen an analytical focus on concrete topics, including wages, employment protection legislation and collective bargaining institutions, rather than a predefined social policy field. Furthermore, Crespy and Vanheuverzwijn point out that although ‘social investment is more represented in the CSRs in proportion to social retrenchment [the latter primarily indicating budget cuts], it remains that the latter often relies on more solid legal foundations’ (2017: 15). This is an important point about equivalent weighting being given to binding and non‐binding CSRs in much of the socialization debate as it stands, something we also address in more detail below.

While we recognize the advances that the socialization literature has provided to our understanding of the functioning of the Semester, there are still limitations present that require attention. The first limitation is the lack of conceptual reflection on what the Semester is. As it stands, the socialization literature seems to accept the EU's own understanding of the Semester without critical engagement. This is problematic, as while the Semester is clearly the main framework for controlling national fiscal and economic policy, the narrow focus only on CSR documents within the literature does not recognize the more encompassing regime of governance instruments available to manage economic and fiscal policy. For instance, when an EU member‐state is subject to a Memorandum of Understanding (MoU), the CSRs for this country simply state that the programme should be implemented. The only CSR for Ireland in 2011, for example, simply states to implement the measures specified in the MoU and its updates. As this CSR was SGP‐ and MIP‐relevant, both the Commission and the Council would have been able to issue financial sanctions for Ireland in case of non‐compliance. The more obvious threat for Ireland, however, was the EU's, IMF's and ECB's ability to withhold the Troika's emergency funding. While creating a parallel process of policy prescription and implementation, Ireland's MoU with the Troika was nevertheless firmly rooted in the institutional framework of the Semester (Marginson 2015: 109).

At present, the socialization literature does not incorporate the policy conditionality of the programmes into their analysis. This is an important omission as it avoids dealing with the problem of how to analyse the new economic governance (NEG) regime as a multi‐faceted but interrelated whole. Can we confidently say that the MoUs on financial assistance and the corresponding economic adjustment programmes (EAPs) are a thing of the past? If so, it would perhaps be justifiable not to include an analysis of the various MoUs as part of this debate. However, we would argue that the EU's financial assistance programmes were not some *ad‐hoc* arrangement that will ultimately give way to relying on the Semester instruments that the socialization debate authors focus on. With the development of the European Stability Mechanism (ESM), alongside continuing strengthening of additional crisis‐fighting tools of the EU,^4^ we must take seriously the need to account for the already broader institutional and legal remit of the Semester that scholars, or even the EU itself, have yet to come to terms with.

The second, but related, issue is that scholars have given equal weighting to policy prescriptions that rest on different legal bases. For instance, there is no differentiation made between the non‐binding CSRs that relate to the EU's Europe 2020 strategy and the binding CSRs that relate either to the SGP or the MIP, which can be enforced, since 2011, by financial sanctions for Euro zone member‐states or, since 2014, by the withdrawal of EU structural and investment funding from all non‐complying EU member‐states. The importance of this point is only exacerbated if we include broader instruments of the NEG regime, such as MoU conditionality. In the analysis below, we therefore do not treat distinct policy prescriptions as the equal of all others. Instead, we analyse the varied levels of constraint that accompany EU prescriptions over each Semester cycle. This will include a focus on the instrument that a prescription is related to, that is, the SGP or MIP, as well as to the level of supervision that a specific state finds itself under, that is, the quarterly reviews regarding an MoU or the in‐depth reviews regarding an excessive deficit or macroeconomic imbalance.

The third important issue is that the socialization literature's analysis of Semester recommendations is wholly dis‐embedded from where the receiving state is situated within the EU's political economy. As it stands, there is simply no attempt to understand, let alone explain, why a specific set of policy prescriptions may be targeted at a specific member‐state at a given point in time, in relation to both their national and the broader European political and economic context, and therefore how this should be factored into our assessment of whether the Semester is becoming socialized or not. Therefore, below, we analyse Semester policy recommendations only in relation to a set of four member‐states as opposed to all 28, but in much more depth; as the analysis of the *transnational* dynamics that are at work here requires a deep knowledge of the affected member‐states and the corresponding language skills (Almond and Connolly 2019; Erne 2018, 2019). Our ‘multi‐sited’ set of inquiry (Marcus 1995) thus includes the EU‐level, two larger countries (Germany and Italy) and two smaller ones (Ireland and Romania) that we know very well. We have chosen to study the transnational NEG system in these locations as they are proxies for the relative power of larger/smaller states and also represent richer/poorer states within the EU's governance regime. This allows us to capture the national and transnational dynamics that are at work in the NEG system.

Collectively, these issues present the need to develop an innovative conceptual and methodological framework to be able to evaluate whether the NEG regime has become progressively socialized since its introduction during 2010 and 2011. The article now turns to detailing our own approach to analysing the Semester prescriptions.

## 3 Studying the European Semester: An alternative analytical approach

Having discussed limitations present across the socialization debate literature, the article now turns to outlining and justifying an alternative approach. Applying this approach will then also allow us to demonstrate its strengths, while providing an original primary analysis of the NEG regime. This analysis will focus on policy prescriptions that are directly relevant for industrial relations, that is, wages, employment protection legislation and collective bargaining institutions. The countries of focus include Germany, Ireland, Italy and Romania. In addition, we also focus on euro area wide documentation. There are several pillars to our analytical approach, which, in turn, address each limitation discussed above.

The first limitation discussed above — the lack of conceptual reflection on what the Semester is — is overcome by including the various MoUs on conditional financial assistance into the analysis, given their inclusion into the NEG process as outlined above. It is important to ensure that there is an accurate reflection of how the Semester functions as a yearly cycle of agenda setting, surveillance, reporting, policy prescription and implementation. The analysis that has been conducted so far within the socialization debate has left a significant sample of data outside of the remit of its analysis.

The second limitation discussed above — equal legal and institutional weighting being given to all policy prescriptions — is addressed twofold. First, when analysing specific policy recommendations, we situate each country in relation to its status within the broader NEG regime. This is just one of the advantages of selecting a smaller cohort of countries to focus on, as the various constraints faced by a member‐state can be taken account of, providing a depth and nuance to our analysis below, which surpasses what has been achieved in other parts of the socialization debate literature thus far.

Table 1 shows the status each country under study was experiencing within the EU's NEG regime between 2009 and 2019. To clarify, there are three constraining EU processes. The first is the SGP, where member‐states can either be within the specified fiscal and debt boundaries that have been approved, or at least on a numerical trajectory towards them, in a significant deviation procedure (SDP) which is a preventative mechanism, or in an excessive deficit procedure (EDP) which is a corrective mechanism. The second NEG process is the MIP. Again, member‐states are either considered to not be experiencing any form of macroeconomic imbalance, which is assessed against a set of macroeconomic indicators, for example, nominal labour unit cost, as well as in‐depth reviews conducted by the Commission, if deemed necessary. Member‐states may experience ‘no imbalances’, ‘imbalances’ (IMB), ‘excessive imbalances’ (Ex‐IMB) or even being placed in a corrective ‘excessive imbalance procedure’ (EIP). Finally, member‐states may be subject to complying with an MoU on financial assistance, which includes full MoU status, whereby conditional support is being provided, or a precautionary MoU (P‐MoU) status, whereby financial support is available if required but is not actually being drawn upon. As mentioned above, member‐states may also receive CSRs in relation to the Europe 2020 strategy on smart, sustainable, inclusive growth. Any non‐implementation of Europe 2020‐related CSRs, however, does not affect a member‐state's status in EU's NEG policy enforcement regime.

**TABLE 1:** Country Status within the EU's NEG Policy Enforcement Regime

	Germany			Ireland			Italy			Romania		
Process	SGP	MIP	MoU	SGP	MIP	MoU	SGP	MIP	MoU	SGP	MIP	MoU
2009	EDP			EDP			EDP			EDP		MoU
2010	EDP			EDP		MoU	EDP			EDP		MoU
2011	EDP			EDP		MoU	EDP			EDP		MoU & P‐MoU
2012^a^				EDP		MoU	EDP	IMB		EDP		MoU & P‐MoU
2013				EDP		MoU		IMB				MoU & P‐MoU
2014		IMB		EDP	IMB			Ex‐IMB				P‐MoU
2015		IMB		EDP	IMB			Ex‐IMB			IMB	P‐MoU
2016		IMB			IMB			Ex‐IMB				
2017		IMB			IMB			Ex‐IMB		SDP		
2018		IMB			IMB			Ex‐IMB		SDP		
2019		IMB			IMB			Ex‐IMB		SDP		

*Source*: Council recommendations on national reform programmes.

^a^ The revised SGP and the new MIP process came into force in 2012.

SGP (Stability and Growth Pact), EDP (Excessive Deficit Procedure); Significant Deviation Procedure (SDP).

MIP (Macroeconomic Imbalance Procedure), Ex‐IMB (Excessive Imbalance), IMB (Imbalance).

MoU (Memorandum of Understanding on Financial Assistance), P‐MoU (Precautionary MoU).

The level of constraint a member‐state is facing within the NEG regime primarily depends on its status in the three NEG enforcement processes, as outlined in Table 1. However, a second step is required to understand how institutional and legal hierarchies are present. Table 2 outlines our attempt to produce such a hierarchy. Column 1 focuses on the origin of different policy prescriptions. Across the three rows, we can see all the various processes that are part of our own definition of what the Semester is. These procedures are then distinguished in such a way that reflects the severity of the possible enforcement mechanisms (columns 2 and 3). For instance, the financial support offered through MoUs can be withdrawn, preventing a member‐state from being able to finance its debt. This is a ‘very significant’ constraint, as a lack of access to alternative funding on financial markets leaves states with few other options than to comply with the programme parameters. On the other extreme, Europe 2020 CSRs can only be enforced through peer pressure, such as naming and shaming or ‘mutual learning’ exercises, which may convince officials from non‐compliant member‐states to change policy (Zeitlin 2016).

**TABLE 2:** Origin and Degree of Constraint of NEG Prescriptions

Origin of Prescription	Enforcement Mechanisms	Coercive Power
**MoU Process** MoU‐ and Precautionary MoU‐related prescriptions	Withdrawal of financial assistance^a^ Withdrawal of EU funding^b^ Financial fines^c^ ^,^ ^d^ Naming and shaming	**Very Significant**
**SGP / MIP Processes** SGP‐ and MIP‐related prescriptions for states with excessive deficits or excessive macroeconomic imbalances	Withdrawal of EU funding^b^ Financial fines^c^ ^,^ ^d^ Naming and shaming	**Significant**
**SGP / MIP Processes** SGP‐ and MIP‐related prescriptions for states with NO excessive deficits or excessive macroeconomic imbalances	Naming and shaming	**Weak**
**Europe 2020 Strategy Process**		
Europe 2020‐related prescriptions		

*Source*: Adapted from Stan and Erne (2018).

^a^ EU Financial Assistance to a member‐state is conditional on the implementation of the corresponding MoU.

^b^ Since 2014, European Structural and Investment funding to all EU member‐states is conditional on ‘sound economic governance’, that is, the implementation of corrective EAP‐, SGP‐, and MIP‐prescriptions (Article 23, Regulation No 1303/2013 of the European Parliament and of the Council of 17 December 2013).

^c^ Since 2011, a member‐state of the euro area that has not ‘taken effective action to correct its excessive [budget] deficit’, risks ‘a fine, amounting to 0.2 per cent of the member‐state's GDP in the preceding year.’ (Art. 6, Regulation No 1173/2011 of the European Parliament and of the Council of 16 November 2011).

^d^ Since 2011, a member‐state of the euro area that ‘has not taken the corrective action [against excessive macroeconomic imbalances] recommended by the Council’ risks an ‘annual fine of 0.1 per cent of the GDP in the preceding year of the member‐state concerned’ (Art. 2, Regulation No 1174/2011 of the European Parliament and of the Council of 16 November 2011).

In order to be able to assess the social trajectory of the NEG process, Table 3 first distinguishes different policy trajectories in three main industrial relations areas^5^ based on NEG prescriptions’ commodifying or decommodifying content. For wage policy, there is a simple division between prescriptions in favour of wage‐level increases and restraints. For labour market institutions, there is a focus on whether there is a call to increase or decrease workers’ employment protections. For collective bargaining institutions, we distinguish between prescriptions that favour solidaristic or individualizing bargaining institutions. We define collective bargaining institutions as solidaristic if they are taking wages and working conditions out of competition through the setting of standards that apply to multiple employers. By contrast, collective bargaining policy recommendations are commodifying and individualizing labour if they call for a decentralization of multi‐employer collective bargaining agreements (Schulten 2002; Stan and Erne 2016).

**TABLE 3:** Policy Trajectories and Themes of NEG Prescriptions

	***Wages***	***Employment Protections***	***Collective Bargaining***
***Decommodification Trajectory***	**Increase wage levels**	**Increase job protection**	**Solidaristic bargaining institutions**
*Themes found in NEG prescriptions*	Sustain wage growth	Facilitate transition to standard employment	Improve social dialogue
	Reinstate national minimum wage		
***Commodification Trajectory***	**Restrain wage levels**	**Decrease job protection**	**Individualizing bargaining institutions**
*Themes found in NEG prescriptions*	Reduce national minimum wages	Ease legislation regulating dismissals	Decentralize collective bargaining
	Monitor effects of minimum wage	Increase the use of fixed‐term contracts	Reform sectoral wage‐setting mechanisms
	Reduce public‐sector wage bill		
	Establish transparent minimum wage‐setting mechanism		

*Source*: Online Annex. Our Analysis of Council Recommendations on National Reform Programmes (2009–19).

Table 3 also synopsizes the emerging themes of the NEG prescriptions of our documentary analysis and their orientation. If one classifies NEG prescriptions simply on their face value, they often appear as ‘ambiguous’ (Crespy and Vanheuverzwijn 2017). However, if one analyses them in the semantic context in which they are situated (Stan and Erne 2018, 2019: 5), their orientation becomes much clearer. Once we understood that the apparently ‘ambiguous’ prescriptions on the ‘establishment of ‘transparent’ minimum wage‐setting mechanism’ was meant to stop a social democratic government from unilaterally increasing minimum wages, for example, it became clear that this prescription was meant to restrain wage increases. Thus, we focus our semantic documentary analysis on instances that we know well.

The third limitation discussed above — failing to account for member‐states’ position within the EU's broader political economy — is addressed by conducting an incorporated comparison of extended case studies (Burawoy *et al*. 2000; McMichael 1990). This approach ensures a more systematic understanding of why specific trajectories are cohering as a relational whole. Therefore, while our focus on CSRs is unavoidable, given the methodological nationalism of the Semester itself, our use of the extended case‐study method allows us to examine how the Semester is connecting different national and supranational sites to each other by focusing on each policy domain first and foremost, collecting data from each set of national and EU documents to provide a detailed understanding of how EU policy prescriptions have evolved over time. If divergence exists between countries regarding the prescriptions they receive, then these will not be related back to some isolated national feature but will be situated in the context of the country's place in the broader uneven and integrated EU political economy.

Having detailed the methodological approach that this article takes, the article now turns to applying these considerations in the next section.

## 4 The EU's labour policy interventions in Germany, Italy, Ireland and Romania (2009–2019)

In this section of the article, we outline the findings from our documentary analysis, which covers over 90 documents, including the Commission's Country Reports, the Council Recommendations on National Reform Programmes (the ‘CSRs’) and the MoUs, including their attendant EAPs and quarterly reviews. We distinguish between documents with monitoring aims (country reports and quarterly reviews) and documents with prescribing aims (CSRs and MoUs). From the latter, we extracted the prescriptions on wages, employment protection legislation and collective bargaining for Ireland, Italy, Germany and Romania between 2009 and 2019, as documented in the Online Appendix for this article.^6^ We begin in 2009, as Romania was forced to sign its first MoU in that year. Although the Romanian state was the least indebted of all states under study (including Germany), its dependence on private foreign creditors (primarily French, German, Greek and Italian banks) forced it to enter into a bailout programme earlier than other states. The results of our analysis are summarized in Table 4 and presented in more detail in the Appendix.

**TABLE 4:** EU Prescriptions on Wages, Employment Protection and Collective Bargaining

Decommodifying					Commodifying				
	DE	IT	IE	RO	DE	IT	IE	RO	
2009								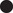	2009
2010							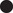 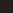	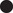	2010
2011			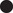			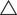 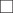	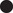 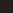	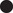 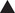 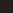	2011
2012	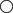					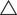 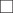	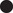 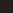	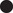 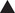 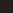	2012
2013	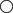 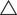					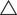 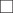	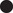 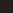	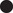 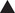 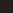	2013
2014	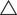				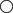	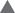 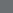		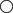	2014
2015	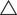					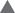 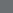		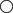	2015
2016	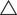							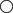	2016
2017	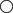 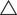					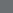		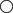	2017
2018	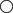			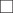				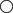	2018
2019	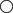							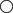	2019

*Source*: Online Annex. Analysis of Council Recommendations on National Reform Programmes.

Thematic Area: Wages (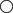 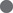 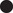); Employment Protection (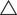 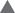 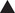); Collective Bargaining (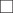 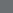 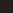)

Degree of Constraints: Very significant (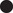 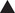 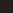); Significant (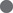 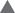 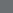); Weak (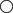 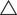 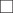).

Table 4 distinguishes between decommodifying and commodifying prescriptions, as outlined in Table 3. Furthermore, Table 4 also distinguishes between very significant (black), significant (grey) and weak (white) prescriptions, based on the different degrees of constraints of a particular NEG prescription depending on its particular policy area, its timing and the country position in the NEG regime, as operationalized in Tables 1 and 2.

At first glance, Table 4 implies that there was no socialization of NEG prescriptions in three fields of labour policy across all four countries under investigation. To understand the meaning of these prescriptions, we must assess them in more detail, taking their time‐ and country‐specific meaning into account.

### Wages

In the area of wages, there are two policy orientations that cohere during the period under study. The first are cuts to wages, particularly across the public sector and cuts to the minimum wage, namely in Ireland and Romania. The second is advocating for an increase in wages for the German economy, particularly across manufacturing sectors. There are no direct prescriptions on wages for Italy. Yet, Italy received prescriptions on labour law and collective bargaining (see below) and on the need to ‘ensure that the general government debt is on a sufficient downward path’ (Council Recommendation 2014/C272/16), which also affected Italian wage developments in the public sector (Bach and Bordogna 2013).

The most pertinent prescriptions on wage restraint fall within the time frame of 2011–2013, when the Euro crisis was acute and when Ireland and Romania were subject to MoU conditionality. The Irish government had already started to implement cuts to public‐sector wages in 2009, in what the IMF defined as one of the most severe austerity programmes in modern times (Whelan 2014). In turn, the Commission's DG ECFIN used the ‘determined policy action’ of the Irish government as an example for others, as the ‘substantial wage adjustment in the public sector in 2009 helped to initiate the necessary change in labour costs’ across all sectors (European Commission 2010: 31 and 67). Even so, the cuts did little to improve the economic situation, and the Irish government was forced to enter a Troika‐led MoU programme in November 2010. As a result, further wage cuts were prescribed, even if Irish nominal unit labour cost (ULC) rates always remained well below the upper ceiling of +9 per cent (over the past three years) set by the NEG regime's own MIP scoreboard. In fact, Irish ULC rates evolved from −2.3 per cent (MIP Scoreboard 2010) to −17.2 per cent (MIP Scoreboard 2017). The wage‐related prescriptions were very precise, often relating to numerically defined targets that had to be met over a specific period. It was only in 2013 that there was a move from cuts to wage moderation in the prescriptions.

In 2014, another theme appeared in the prescriptions on wages for Romania, namely calls for ‘objective’ criteria when establishing minimum wage levels. As the (explanatory) recitals of the corresponding Council Recommendations include a reference in favour of social dialogue,^7^ analysis that does not take account of the local context could interpret them as being ‘social’. However, if placed into context, one realizes that they were meant to restrain wage increases. The prescriptions were directed against the new social democratic government who promised to counter the wage cuts suffered by Romanian workers during the crisis by (unilateral) minimum and public‐sector wage increases. Although the minimum wage increases since 2013 did not undermine the international competitiveness of Romanian firms (Heemskerk et al. 2018), the government adopted in 2017 a radical tax reform that shifted the burden of almost all social insurance taxes from employers to employees. With this move, the government attempted to ‘avoid a hike in the public deficit’ caused by the public sector and minimum wage increases it scheduled for 2018 (Stoiciu 2018a). It is noteworthy that this tax revolution was implemented just after the EU opened an SDP against Romania. Although Romania still had one of the lowest public debt to GDP ratios in the EU (37.5 per cent in 2017), the Council asked the Romanian government to take decisive action to ensure that the nominal growth rate of net primary government expenditure would not exceed 3.3 per cent in 2017 (Council Recommendation, 16 June 2017, C 216/01).

A similar development occurred in Ireland in 2011, when the Troika and the new Irish government agreed to reinstate the statutory minimum wage at its original level, following also a high‐profile industrial dispute (Hickland and Dundon 2016). The corresponding MoU stated: ‘We will reverse the recent reduction in the national minimum wage, mitigating any effects on employment through the targeted reduction in PRSI [Pay‐Related Social Insurance] (Ireland, MoU, 1^st^ update, 28/04/2011)’.

The second major orientation that coheres in the analysed NEG documents is concerned with pursuing a sustained increase in wages across the German economy. It is important to note that the degree of constraint is non‐binding across all relevant prescriptions, with a lack of numerical precision, apart from reference to the Keynesian ‘Golden Wage Rule’, which incidentally has inspired European trade unions’ wage bargaining coordination efforts since 1999 (Erne 2008). Most of the discussion also falls outside of the Council's CSRs, which is not surprising given the strong political resistance in the so‐called surplus countries against criticisms of their wage policies (Bieler and Erne 2014). These criticisms point to a perception that German policy makers favoured ‘beggar‐thy‐neighbour’ wage policies, generating a high degree of export competitiveness, but at the expense of its EU partners (Flassbeck and Lapavitsas 2013). Prescribing an increase in wages is thus not only aimed at restructuring the German economy, but also about the way that such a restructuring will create spillover effects for the capability of economic operators in other EU member‐states to recover. Although the tentative calls for higher German wage increases were welcomed by unions across Europe, it is equally clear that on balance the wage constraining prescriptions prevailed across time and our sites of inquiry.

### Employment Protection Legislation

In the area of employment protection, there are again two major policy orientations that cohere. The first seeks the removal of labour market ‘rigidities’ and an increase in ‘flexibility’. In other words, there is a persistent call that the economic risk should shift from firms to workers. This is evident across documentation for the Euro Area as a whole, as well as Ireland, Italy and Romania.

It is no surprise that throughout euro area documentation, there is little precision in the language used about how exactly greater flexibility and reduced rigidity should be achieved for labour markets. The documentation, however, also regularly states the need to pursue these aims in line with principles of flexicurity. Scholars advocating for the thesis that we have witnessed the socialization of the Semester, such as Sonja Bekker, point to a revitalized use of the flexicurity approach as a key piece of evidence, even going so far to argue that its definition has been widened to encompass a larger range of security issues than were covered previously (Bekker 2017). While in this study, we also find a call for principles of flexicurity to be respected when implementing labour market restructuring, these appear inconsistently, are most explicit in documentation that has no binding force on member‐states and lack any precision of language about how this could be achieved. This is hardly surprising given ‘the opposing viewpoints’ that flexicurity attempts to reconcile in one catchphrase (Hyman 2005: 25).

When it comes to flexicurity, it is only in the case of Romania where this concept is explicitly employed, and this is always in an imprecise manner. The MoU signed in 2011 states, ‘improve the adequacy of the employment protection legislation and adapt to the flexicurity principles’ (P‐MoU, 28/06/2011). There are also explicit references to what improving the ‘adequacy’ of legislation actually means, including ‘widen the set of cases for use of fixed‐term labour contracts’ (P‐MoU, 1^st^ Amendment, 27/12/2011). Here, clearly, the need for flexibility is being prescribed. However, there are no equivalent measures targeting improved security for workers to convincingly argue that the two components of the concept are being fulfilled. It is also important that calls for liberalization are being made in the MoU that is binding on Romania. In turn, the Romanian government used emergency law to push through a major liberalizing labour law reform, despite a relatively high union density and union protests, notably in the public sector (Adăscăliței and Muntean 2019; Stan and Erne 2016).

There is a similar focus on flexibility across the prescriptions for Italy. Principally, EU‐level documentation is concerned with ensuring that it is easier for Italian firms to dismiss workers. This would act as both a means to ensure that firms are not burdened with unnecessary labour during periods of crisis and to encourage firms to hire more people in good times. This strategy is specifically aimed at addressing ‘labour market segmentation’ and the lack of employment opportunities for young workers. For instance, the relevant 2011 CSR states, ‘reinforce measures to combat segmentation in the labour market, also by reviewing selected aspects of employment protection legislation including the dismissal rules and procedures’ (Council CSR 2011/C 215/02, 12/07/2011). When corresponding legislation was brought forward by the Italian government in 2012, there was a strong EU approval of the package, highlighting the concern with moving towards greater flexibility.

Following the adoption of further liberalizing legislation in 2015/6, EU‐level documentation reinforces the argument that it is primarily concerned with flexibility, as the 2016 Country Report states: ‘the revision of the rules for unfair dismissal increases exit flexibility and substantially increases legal certainty’ (CR SW(2016) 81 final, 26/02/2016). While there is a lack of precision in the language adopted throughout EU‐level documentation, leaving it to the Italian government to define the exact focus of the legislation to be presented — something quite different from the extremely detailed prescriptions that are a feature of MoU documentation — the constraints on the government do increase over the period under study, given the shift in Italy's status from having imbalances to having ‘excessive imbalances’.

When the Troika arrived in Ireland at the end of 2010, the Irish labour market was already one of the most deregulated among OECD countries. Yet, ‘labour market flexibility’ was cited as a compelling reason to liberalize the only existing sectoral regulations on workers’ terms and conditions, which we address in the following section (Ireland, EAP, Autumn 2011 Review: 32–33). After the Troika left the country at the end of 2013, there has been no call within the Semester to increase Irish employment protection legislation.

Again, it is only in the case of Germany that there are a set of policy concerns distinct from the dominant trend for the other three countries. First, it is worth pointing out that there is a persistent call for the German government to support workers to pursue a transition away from ‘mini‐jobs’ to more traditional forms of employment; the former being a highly flexible form of employment that places strict limits on the income that can be earned and excludes social security payments being made. Having emerged out of the Hartz reforms introduced in 2003 to address concerns over high unemployment (Bruff 2010), mini‐jobs have grown to include several million workers. It is here where there is a greater concern for workers’ security being demonstrated, when compared with the other documentation analysed. However, if the growth of increasingly flexible forms of employment is a real concern for EU‐level institutions, then why are reforms to increase flexibility at a rapid pace being prescribed for other countries?

### Collective Bargaining

The dominant reforms being called for in the area of collective bargaining fit within the broader strategy of achieving economic recovery through the logic of ‘internal devaluation’. There are persistent calls for bargaining institutions to be decentralized to firm level, particularly across Ireland, Italy and Romania, to enable wage adjustments that better reflect the productivity development of companies.

This is evident in the case of Romania, which during the period of the financial assistance programme received prescriptions to ‘implement reforms to the wage‐setting system allowing wages to better reflect productivity developments in the medium term’ (Romania, MoU, 28/6//2011). In turn, the national and sectoral wage bargaining institutions were subjected to a ‘frontal assault’ in the form of another emergency law adopted by the centre‐right Romanian government (Trif 2016). After a subsequent social democratic government unilaterally increased minimum wages, however, it was told in 2018 to refrain from further unilateral action and to strengthen social dialogue (Council CSR 2018/C 320/22, 13/07/2018).

In 2011, the then chairman of the ECB, Trichet and his successor Draghi, also asked the Italian government in a leaked letter to ‘reform the collective wage bargaining system allowing firm‐level agreements to tailor wages and working conditions to firms' specific needs’ (*Corriere della Sera*, 5/8/2011). This message frequently reappeared in several CSRs Italy receives over the years. This created continuous pressure towards a further decentralization of collective bargaining to the firm level, even if multi‐employer agreements at the sectoral level still play a notable role in Italy (Regalia and Regini 2018), in contrast to the Romanian and Irish cases.

The reform of sectoral wage‐setting mechanisms, which became part of the Irish programme agreed with the Troika, was also justified on the ground that it should have ensured ‘wages are adequately linked to [firm‐level] productivity levels’ (Ireland, EAP, Autumn 2012 Review: 37–38). The Irish case constitutes an interesting example of change in the discourse orientation of the policy prescriptions. At the beginning of the Irish programme, the first MoU only required a review of the sectoral institutions. Yet, once two successive Irish courts’ judgments struck out sectoral wage setting as unconstitutional, the prescriptions became increasingly precise and targeted during the reform process which followed (Maccarrone *et al*., 2019). Once again, the absence of similar calls since 2014 in favour of collective bargaining decentralization does not constitute an example of ‘socialization’, but simply reflects, on the one hand, the fact that the requests contained in the MoUs were implemented, and, on the other hand, that the legislation protecting and enforcing collective bargaining rights was already remarkably weak.

### Contextualized Comparative Discussion

Our analysis shows that there has been no socialization of the NEG regime between 2009 and 2019 in the policy fields and countries under study. While the coercive strength of commodifying prescriptions decreased over time, the Italian and Romanian governments continued to receive commodifying prescriptions well after the start of the economic recovery. In line with the long‐standing liberalization trend of industrial relations (Baccaro and Howell 2017), there was a continuing insistence on both the commodification of job protection laws and wage bargaining decentralization for Italy, despite the implementation of several radical reforms from 2011 to 2015 (Rutherford and Frangi 2018). In 2018, however, the issue of bargaining decentralization was dropped from the prescriptions. But this did not happen due to a higher sensitivity to social or local concerns, as the Commission continued to call the efforts on decentralization insufficient (Commission, Country Report 2018, Italy). The recommendation was dropped only after the Employment Committee of the Council evaluated the level of decentralization achieved by the Italian reforms as sufficient (EMCO, Thematic Review, 25/01/2018). At times ‘local context’ does indeed count (Pochet 2019: 286), but only if recognized by at least 16 Council delegations from member‐states representing at least 65 per cent of the total EU population. The uneven implementation of CSRs, as measured by the Commission itself (Al‐Kadi and Clauwaert 2019), therefore hardly represents a cause for respite for labour movements. Governments can never be sure ‘in advance whether or not their “reform program” will satisfy the EU executives. The ambiguous grounds for sanctions therefore represent a risk that policymakers find difficult to assess’ (Erne 2015: 347). This point especially applies to small, dependent market economies like Romania (Ban 2019).

The MoU prescriptions for Romania called for major wage cuts and a commodification of individual and collective labour law. The 2011 law on ‘Social Dialogue’ abolished national, multi‐employer bargaining by law and increased unions’ representativeness thresholds to a level that deprived most of them of their bargaining rights. This would suggest that there would be no need for further *new* prescriptions after the acute phase of the crisis. In 2013, however, the EU started to worry about the increases in public sector and minimum wages that the new social democratic government promised to implement unilaterally. First, the CSRs that were meant to prevent those increases had no effect. In 2017, however, the Council opened an SDP that meant that the government had to take decisive action to ensure that the nominal growth rate of net primary government expenditure would not exceed 3.3 per cent in 2017. In turn, the social democratic government counteracted its own wage increases by a new law that shifted most social security taxes of employers to their employees. Since 2018, its public sector and minimum wage increases have thus effectively been financed by the employees themselves, given the savings for both public and private employers created by this ‘tax revolution’ (Stoiciu 2018a). In every third officially recorded employment relationship, net earnings even decreased despite high economic growth rates (Stoiciu 2018b).

In Ireland, the MoU also called for major wage cuts and liberalizations of its already very flexible collective wage‐setting regime. After the abolishment of the provisions for binding sectorial minimum wages and their replacement by a more flexible regime in only a small number of sectors, Ireland did not receive any commodifying CSRs in our fields of observation, as successive Irish governments made sure that the austerity wage cuts were restored at such a slow pace that they did not cause any concerns in Brussels. Irish growth rates also increased again, not as a result of the austerity cutbacks, but due to the growth of actual and transfer pricing activities that multinational firms reported in Ireland. In turn, the Irish nominal ULC increases for the 2014–2016 period remained a stunning 29.5 per cent below the ceiling set by the EU's MIP scoreboard (Commission, Alert Mechanism Report 2018, COM(2017)771).

The German government, by contrast, received weak prescriptions that point in a decommodifying direction. Since 2013, Germany has faced persistent calls to increase wages. This is, however, not due to the German economy being besieged by forms of employment that are paid lower than equivalent forms of work elsewhere, but because of its position within the EU economy. According to EU policy makers, German wage policy alone would be able to generate so much demand‐led economic growth domestically that it would have positive ‘spill‐over effects’ for the rest of the EU (Buti and Turrini 2017). What on the surface looks like a shift towards social concerns about the wage moderation that has been forced upon German workers for an extended period, is, in fact, an economic concern with the role current account imbalances have played in shaping macroeconomic trajectories across the EU.

## 5 Conclusion

As the making of the EU market did not lead to economic convergence but to crisis‐inducing imbalances, adjustments had to be imposed by fiat. After the crisis shattered beliefs in self‐regulating markets, national industrial relations became subject to ‘vertical’ EU governance interventions (Erne 2018). The turn to the NEG regime has already been problematized in theory. There are also numerous case studies on the social consequences of this shift. Others have tried to capture the nature of NEG by the counting and coding of hundreds of CSRs (Copeland and Daly 2018; Crespy and Vanheuverzwijn 2017; Zeitlin and Vanhercke 2018). However, what has been missing is an analysis of NEG prescriptions across a smaller sample of countries to allow meaningful, contextualized comparisons. We studied policy prescriptions on industrial relations issues for the eurozone as a whole and for two larger and two smaller countries. While country size is a proxy for political influence in the EU, our selection also includes places that represent different locations across the EU's asymmetrical political economy. Our focus on four countries also allowed us to take the different contexts and constraints of prescriptions into account. This approach has distinguished our analysis from the decontextualized counting exercises that treat all CSRs equally. This matters as our distinct approach has led us to different results.

Our incorporated comparisons of the EU's NEG prescriptions for Germany, Italy, Ireland and Romania since 2009 show that there has been no socialization in the three fields of industrial relations under investigation. Although the constraining power of commodifying CSRs declined over time, it would be wrong to dismiss the NEG regime as ‘soft governance’ (Pochet 2019: 282). Our analysis shows that the easing of constraints does not denote a policy shift within the Semester, but rather the implementation of major commodifying reforms in Italy, Ireland and Romania during the crisis years and the declining constraining force of CSRs in times of economic recovery. But make no mistake, the NEG regime institutionalized in 2011 is likely to come back with full force in the next crisis if it is not altered in time. Our findings therefore do not support those that regard the Semester as a benign process that has become socialized.

At the same time, looking ahead, there are also internal contradictions that may be exploited by labour in their favour. Unions could use the references to the Keynesian ‘Golden Wage Rule’ in the 2016 Country Report for Germany as an argument for a more expansionary wage policy. After all, EU peer pressure on wages has helped unions in Germany in achieving higher wage increases (Lübker 2019: 19), which is also in the interest of workers elsewhere. Furthermore, unions could try to turn the EU's own ULC benchmarks on its head, given that the MIP scoreboard ceiling for acceptable wage increases (plus 9 per cent in the eurozone and plus 12 per cent in non‐Euro area) has been reached almost nowhere (Commission, Alert Mechanism Report 2019, COM/2018/758 final). After all, in times of recovery, European employers and policy makers may also rediscover the merits of ‘social dialogue’ that they banished in the crisis (Buti and Turrini 2017). The new Commission President von der Leyen also said in her candidacy speech that ‘every person that is working full time should earn a minimum wage that pays for a decent living’ and promised to create a framework to achieve this (Commission, SPEECH/19/4230). Conversely, however, the current debate around the eurozone budget also shows NEG's continued commodifying bias, as the proposed budget has no redistributive function, but would serve to reward states that implement ‘structural reforms’ (Council of the European Union 2019).

Most importantly, however, in the absence of labour mobilizations, there is no need for social concertation, which means that even moderate unions must complement the force of their arguments with the argument of force (Bieler *et al*. 2019). Given methodological nationalism of NEG, its technocratic language and the different direction and cohesive power of the analysed industrial relations prescriptions for countries that are located in different places in the NEG system, it is not easy for unions to politicize NEG in a transnational public sphere (Erne 2015). Considering the much more uniform commodification patterns of CSRs on the provision of public services (Stan and Erne 2019; Szabó 2019), however, NEG may be politicized more easily by European public service unions rather than European manufacturing unions that faced ‘challenges that transcend the national level’ for much longer (Vulkan and Larsson 2019: 158).

## Supporting information

Online Appendix

## Notes

1 Roland Erne, Participant Observation. ‘Social Dialogue Actors and [Troika] Institutions in the Context of Deepening EU Integration’, round table at the ILO/European Commission seminar: *The governance of policy reform in Ireland*, Dublin Castle, 7 December 2012.

2 ‘It is unacceptable to me that workers and retired people had to shoulder the burden of structural reform programmes, while ship owners and financial speculators became even richer. In the future we need a more democratically legitimate replacement for the Troika and thorough social impact assessments for any new support programmes’ (Juncker 2014).

3 The high‐level contacts between the scientific promoters of the ‘socialization’ thesis and the EU policy making community are also worth noting. According to the economic adviser responsible for the Euro and Social Dialogue in President Juncker's cabinet, these contacts date back to the creation of the Lisbon agenda (Tholoniat 2010: 95).

4 See the European Financial Stability Mechanism and the European Financial Stability Facility as well as the Regulation No. 472/2013 of the European Parliament and the Council on 21 May 2013 on the ‘strengthening of economic and budgetary surveillance of Member states in the euro area experiencing or threatened with serious difficulties with respect to their financial stability’.

5 We have therefore excluded the NEG prescriptions on pensions and other welfare payments, on the introduction of performance related pay and other HRM systems in the public sector as well as on the reduction of payroll taxes from our analysis.

6 All NEG prescriptions for Germany, Italy, Ireland and Romania (2009–2019) on wage policy, employment protection legislation and collective bargaining have been coded by policy orientation and their enforcement power, as documented in the Online Appendix.

7 ‘The minimum wage, which is among the lowest in the Union, has increased substantially since 2013 and the lack of objective criteria for its setting creates uncertainty. A tripartite working group has been set up to work on the reform of minimum wage setting, but there continues to be no clear guidelines or criteria that would take into account its impact on job creation, social conditions and competitiveness. Overall, social dialogue remains weak.’ (Recital 12, Council Recommendation 2016/C 299/189).
