# Supplementary material for: Towards a Socialization of the EU's New Economic Governance Regime? EU Labour Policy Interventions in Germany, Ireland, Italy and Romania (2009–2019)
Source: Br J Ind Relat. 2020 Feb 18;59(1):191–213. doi: 10.1111/bjir.12522 (PMC7891581; doi:10.1111/bjir.12522)
Supplement: Supplementary file 1 — Online Appendix [file BJIR-59-191-s001.docx]

**Online Appendix -- Data Tables**

**New Economic Governance Prescriptions (NEG) for Germany, Italy, Ireland and Romania (2009-2019) on Wage Policy, Employment Protection Legislation and Collective Bargaining by policy orientation and enforcement power**

**Appendix Table 1a) NEG Prescriptions on Wage Policy: Germany**

| Policy themes | Sustain wage growth | Monitor effects of minimum wage on employment |
| --- | --- | --- |
| 2009 |  |  |
| 2010 |  |  |
| 2011 |  |  |
| 2012 | Create the conditions for wages to grow in line with productivity (Council Recommendation 11255/12, 06/07/2012) **W_INC W** |  |
| 2013 | Sustain conditions that enable wage growth to support domestic demand. (Council Recommendation 2013/C 217/09, 09/07/2013) **W_INC W** |  |
| 2014 |  | When implementing the general minimum wage, monitor its impact on employment. (Council Recommendation 2014/C 247/05, 08/07/2014) **W_RES W** |
| 2015 |  |  |
| 2016 |  |  |
| 2017 | Create conditions to promote higher real wage growth, respecting the role of the social partners. (Council Recommendation 2017/C 261/05, 11/07/2017) **W_INC W** |  |
| 2018 | Create conditions to promote higher wage growth, while respecting the role of the social partners. (Council Recommendation 2018/C 320/05, 13/07/2018) **W_INC W** |  |
| 2019 | Strengthen the conditions that support higher wage growth, while respecting the role of the social partners. (Council Recommendation 2019/C 301/05, 09/07/2019) **W_INC W** |  |

**Appendix Table 1b) NEG Prescriptions on Wage Policy: Italy**

N.A. There are no NEG prescriptions in this field for Italy.

**Appendix Table 1c) NEG Prescriptions on Wage Policy: Ireland**

| Policy themes | Reduce public sector wage bill | Reduce, then reinstate the NMW |
| --- | --- | --- |
| 2009 |  |  |
| 2010 | Adjust… the overall public service wage bill to compensate for shortfalls in savings. (Ireland, MoU, 28/11/2010) **W_RES V**  New public sector entrants will see a 10% pay reduction. (Ireland, MoU, 28/11/2010) **W_RES V** | Reduce by €1,00 per hour the nominal level of the current national minimum wage. (Ireland, MoU, 28/11/2010)  **W_RES V** |
| 2011 | … appropriate adjustment measures, including to the overall public service wage bill, to ensure consistency with the fiscal adjustment targets over the programme horizon. (Ireland, MoU, 1^st^ update, 28/04/2011) **W_RES V**  New public service entrants will also see a 10% pay reduction. (Ireland, MoU, 1^st^ update, 28/04/2011) **W_RES V** | We will reverse the recent reduction in the national minimum wage, mitigating any effects on employment through the targeted reduction in PRSI in the Jobs Initiative. (Ireland, MoU, 1^st^ update: 28/04/2011)  **W_INC V** |
| 2012 | Reduction in the total pay and pensions bill (Ireland, MoU, 4^th^ update, 01/03/2012) **W_RES V** |  |
| 2013 | Reduction in the total pay and pensions bill. (Ireland, MoU, 7^th^ update, 25/01/2013) **W_RES V** |  |
| 2014 |  |  |
| 2015 |  |  |
| 2016 |  |  |
| 2017 |  |  |
| 2018 |  |  |
| 2019 |  |  |

**Appendix Table 1d) Prescriptions on Wage Policy: Romania**

| Policy themes | Reduce public sector wage bill | | Establish ‘transparent’ mechanisms for minimum wage setting |
| --- | --- | --- | --- |
| 2009 | A gradual reduction of the fiscal deficit is envisaged (…). The adjustment will be mainly expenditure-driven, by reducing the public sector wage bill, cutting expenditure on goods and services, lowering subsidies to public entities and through reductions of capital spending on items like vehicles and office equipment. (Romania, MoU, 23/06/2009) **W_RES V**  Establishment of a unified, simplified pay scale and reform the current system of bonuses. This legislation will foresee that the share of the base salary as a percentage of total public compensation should be at least 70%. This will be done through the elimination of the large majority of bonuses or rolling them into the base wage. The creation of non-monetary bonuses will be prohibited. For any given public servant, total bonuses will be legally capped. All bonuses will continue to be fully taxable. The law could provide a phasing-in period of the reforms of up to 3 years. (Romania, MoU, 23/06/2009) **W_RES V** | |  |
| 2010 | Reduction in the public wage bill to 8.7% of GDP in 2010. To this end, nominal wages should be frozen at the level reached by end-2009 (except for minimum public wage earners). (Romania, MoU, 1st addendum, 22/02/2010) **W_RES V**  Introduce expenditure ceiling on public sector wage bill (Romania, MoU, 1st addendum, 22/02/2010) **W_RES V**  A 25% reduction in the public sector wages, bonuses and other compensation paid to all public sector employees from 1 July 2010 onwards, while providing a minimum wage of 600 RON. (Romania, MoU, 2^nd^ addendum, 02/08/2010) **W_RES V**  Abolition of the 2010 thirteenth month salary for public sector employees. This will show up in the 2011 budget. (Romania, MoU, 2^nd^ addendum, 02/08/2010) **W_RES V** | |  |
| 2011 | Enactment of the Unified Wage Law (framework law and implementing law for 2011) with a view to: i) introducing a unitary salary system for the employees working for budgetary entities who are paid by the general government; and ii) limiting the public sector wage bill in 2011 to RON 39 billion. (Romania, MoU, 3^rd^ addendum, 19/01/2011) **W_RES V**  Continued monitoring of the public sector wage bill, and necessary action taken in a timely fashion if wage bill is projected by the MOPF or by the Commission staff to exceed this limit, such that it remains capped at 39 billion RON in 2011 (excluding 1574 million RON in social security contributions for the military as a result of the application of the new pension law). The public sector wage bill should remain sustainable over the 2012-2014 period and respect the relevant limits set in the Medium-Term Fiscal Strategy. (Romania, P-MoU, 29/06/2011) **W_RES V** | |  |
| 2012 | Continued monitoring of the public sector wage bill, and necessary action taken in a timely fashion if the wage bill is projected… to exceed the relevant limits set in the Medium-Term Fiscal Strategy. (Romania, P-MoU, 2^nd^ addendum, 29/06/2012) **W_RES V** |  | |
| 2013 | The public sector wage bill will need to stay on a sustainable footing, limiting wage growth as well as public sector employment levels. (Romania, P-MoU, 06/11/2013) **W_RES V**  Preserve the achievements of the previous two programmes, implement the further measures agreed under those programmes, and fulfil any remaining parts of yet unfulfilled conditionality. This applies in particular to: i) the Unified Wage Law (and the related suppression of the “stimulente"); ii) the medium-term sustainability of the public sector wage bill. (Romania, P-MoU, 06/11/2013) **W_RES V** |  | |
| 2014 |  | Establish, in consultation with social partners, clear guidelines for transparent minimum wage setting, taking into account economic and labour market conditions. (Council Recommendation 2014/C 247/21, 08/07/2014) **W_RES W** | |
| 2015 |  | Establish, in consultation with the social partners and in accordance with national practices, clear guidelines for setting the minimum wage transparently. (Council Recommendation 2015/C 272/01, 14/07/2015) **W_RES W** | |
| 2016 |  | Establish, in consultation with social partners, objective criteria for setting the minimum wage. (Council Recommendation 2016/C 299/18, 12/07/2016) **W_RES W** | |
| 2017 |  | Establish a transparent mechanism for minimum wage setting, in consultation with social partners. (Council Recommendation 2017/C 261/22, 11/07/2017) **W_RES W** | |
| 2018 |  | Ensure minimum wage setting based on objective criteria.(Council Recommendation 2018/C 320/22, 13/07/2018) **W_RES W** | |
| 2019 |  | Ensure that the minimum wage is set on the basis of objective criteria, consistent with job creation and competitiveness. (Council Recommendation, 2019/C 301/23, 09/07/2019) **W_RES W** | |

**Appendix-Table 2a: Prescriptions on Employment Protection Legislation for Germany, Italy and Romania**

| Policy themes | Germany:  Facilitate transition to standard employment | Italy:  Ease legislation regulating  dismissals | Romania:  Increase the use of fixed-term contracts and make use of flexicurity principle |
| --- | --- | --- | --- |
| 2009 |  |  |  |
| 2010 |  |  |  |
| 2011 |  | Reinforce measures to combat segmentation in the labour market, also by reviewing selected aspects of employment protection legislation including the dismissal rules and procedures and reviewing the currently fragmented unemployment benefit system taking into account the budgetary constraints. (Council Recommendation 2011/C 215/02, 12/07/2011) **LM_DEP W** | Widen the set of cases for use of fixed-term contracts (by Autumn 2011), while ensuring that this does not increase labour market segmentation. In parallel, improve the adequacy of the employment protection legislation and adapt to the flexicurity principles. (Romania, P-MoU, 29/06/2011) **LM_DEP V** |
| 2012 |  | Adopt the labour market reform as a priority to tackle the segmentation of the labour market and establish an integrated unemployment benefit scheme. (Council Recommendation 11259/12, 06/07/2012) **LM_DEP W** | Widen the set of cases for use of fixed-term contracts (by end-October 2012), while ensuring that this does not increase labour market segmentation. In parallel, improve the adequacy of the employment protection legislation and adapt to the flexicurity principles. (Romania, P-MoU, 2^nd^ addendum, 29/06/2012) **LM_DEP V** |
| 2013 | Facilitate the transition from non-standard employment such as mini-jobs into more sustainable forms of employment. (Council Recommendation 2013/C 217/09, 09/07/2013) **LM_IEP W** | Ensure the effective implementation of the labour-market and wage-setting reforms to allow better alignment of wages to productivity. (Council Recommendation 2013/C 217/11, 09/07/2013) **LM_DEP W** | Preserve the achievements of the previous two programmes, in particular by monitoring the implementation of the Labour Code and Social Dialogue Code and ensuring that any further amendment to labour legislation will be undertaken in consultation with all stakeholders through ordinary legislative procedures. (Romania, P-MoU, 06/11/2013) **LM_DEP V** |
| 2014 | facilitate the transition from mini‐jobs to forms of employment subject to full mandatory social security contributions. (Council Recommendation 2014/C 247/05, 08/07/2014) **LM_IEP W** | Evaluate, by the end of 2014, the impact of the labour market and wage-setting reforms on job creation, dismissals' procedures, labour market duality and cost competitiveness, and assess the need for additional action. (Council Recommendation 2014/C 247/11, 08/07/2014) **LM_DEP S** |  |
| 2015 | Revise the fiscal treatment of mini-jobs to facilitate the transition to other forms of employment. (Council Recommendation 2015/C 271/01, 14/07/2015) **LM_IEP W** | Adopt the legislative decrees on the design and use of wage supplementation schemes, the revision of contractual arrangements, work-life balance and the strengthening of active labour market policies. (Council Recommendation 2015/C 272/16, 14/07/2015) **LM_DEP S** |  |
| 2016 | facilitate the transition from mini-jobs to standard employment. (Council Recommendation 2016/C 299/05, 12/07/2016) **LM_IEP W** |  |  |
| 2017 | facilitate transitions to standard employment. (Council Recommendation 2017/C 92/01, 21/03/2017) **LM_IEP W** |  |  |
| 2018 |  |  |  |
| 2019 |  |  |  |

**Appendix Table 2b) Prescriptions on Employment Protection Legislation for Ireland**

N.A. There are no NEG prescriptions in this field for Italy.

**Appendix Table 3a) Prescriptions on Collective Bargaining for Germany**

N.A. There are no NEG prescriptions in this field for Germany

**Appendix Table 3b): Prescriptions on Collective Bargaining for Italy and Ireland and Romania**

| Policy themes | Italy:  Decentralise collective bargaining | Ireland:  Reform sectoral wage-setting mechanism |
| --- | --- | --- |
| 2009 |  |  |
| 2010 |  | Government will introduce legislation to reform the minimum wage in such a way as to foster job creation, notably for categories at higher risk of unemployment, and prevent distortions of wage conditions across sectors associated with the presence of sectoral minimum wages in addition to the national minimum wage. Measures will be as follows: (…) An independent review of the Framework REA and ERO arrangements will be initiated by the end of Q1 2011. Terms of Reference and follow-up actions will be agreed with European Commission Services. (Ireland, MoU, 28/11/2010) **CB_IND V** |
| 2011 | Take further steps, based on the 2009 agreement reforming the collective bargaining framework and in consultation with the social partners in accordance with national practices, to ensure that wage growth better reflects productivity developments as well as local and firm conditions, including clauses that could allow firm level bargaining to proceed in this direction. (Council Recommendation 2011/C 215/02, 12/07) **CB_IND W** | Government will discuss with European Commission Services the main findings of the independent reviews of Registered Employment Agreements (REAs) and Employment Regulations Orders (EROs) arrangements, and present a time-bound comprehensive action plan to follow up on its recommendations, taking into consideration the implications of the 6 July 2011 High Court ruling. The ruling found that sections of legislation governing wage-setting mechanisms in EROs are unconstitutional (Ireland, MoU, 2^nd^ update, 28/04) **CB_IND V** |
| 2012 | Monitor and, if needed, reinforce the implementation of the new wage-setting framework in order to contribute to the alignment of wage growth and productivity at sector and company level. (Council Recommendation 2012/C 11259/12, 06/07/12) **CB_IND W** | Building on the Industrial Relations (Amendment) Bill 2011, the authorities will present amendments to the Dáil in particular to: (i) provide that the inability to pay clause for EROs and REAs can allow two consecutive exemptions within the overall two year time limit where this is necessary to safeguard employment, and (ii) ensure that the process for the granting of a variation to an REA is conducted in a timely manner. (Ireland, MoU, 5^th^ update, 25/06/) **CB_IND V** |
| 2013 | Ensure the effective implementation of the labour market and wage-setting reforms to allow better alignment of wages to productivity. (Council Recommendation 2013/C 217/11, 09/07/2013) **CB_IND W** | The authorities will report to the staff of the European Commission, the IMF, and the ECB on the impact on the labour market of reforms to sectoral wage-setting mechanisms undertaken in the programme. (Ireland, MoU, 7^th^ update, 25/01/2013) **CB_IND V** |
| 2014 | Evaluate, by the end of 2014, the impact of the labour market and wage-setting reforms on job creation, dismissals' procedures, labour market duality and cost competitiveness, and assess the need for additional action. (Council Recommendation C 247/11) **CB_IND S** |  |
| 2015 | Promote, in consultation with the social partners and in accordance with national practices, an effective framework for second-level contractual bargaining. (Council Recommendation 2015/C 272/16) **CB_IND S** |  |
| 2016 |  |  |
| 2017 | With the involvement of social partners, strengthen the collective bargaining framework to allow collective agreements to better take into account local conditions. (Council Recommendation C 261/11, 11/07) **CB_IND S** |  |
| 2018 |  |  |
| 2019 |  |  |

**Appendix Table 3c: Prescriptions on Collective Bargaining for Romania**

| Policy themes | Decentralise collective bargaining | Improve functioning of social dialogue |
| --- | --- | --- |
| 2009 |  |  |
| 2010 |  |  |
| 2011 | Implement reforms to the wage-setting system, allowing wages to better reflect productivity developments in the medium term, while respecting the autonomy of social partners, national traditions and practices. (Romania, MoU, 29/06/2011) **CB_IND V** |  |
| 2012 | Implement reforms to the wage-setting system, allowing wages to better reflect productivity developments in the medium term, while respecting the autonomy of social partners, national traditions and practices. (Romania, Supplemental MoU, MoU, 2^nd^ addendum, 29/06/2012)  **CB_IND V** |  |
| 2013 | Preserve the achievements of the previous two programmes, particularly by monitoring the implementation of the Labour Code and Social Dialogue Code. Ensure that any further amendment to labour legislation will be undertaken in consultation with all stakeholders through ordinary legislative procedures. (Romania, MoU, 06/11/2013) **LM_DEP V** |  |
| 2014 |  |  |
| 2015 |  |  |
| 2016 |  |  |
| 2017 |  |  |
| 2018 |  | Improve the functioning of social dialogue.  (Council Recommendation 2018/C 320/22, 13/07/2018)  **CB_SOL_W** |
| 2019 |  |  |

**Appendix-Table 4: Coding Keys for the NEG Prescriptions based on their policy orientation and enforcement power**

| **Wages** | **W_INC W** | Wage Increase, Weak enforcement power |
| --- | --- | --- |
|  | **W_INC S** | Wage Increase, Significant enforcement power |
|  | **W_INC V** | Wage Increase, Very significant enforcement power |
|  | **W_RES W** | Wage Restraint, Weak enforcement power |
|  | **W_RES V** | Wage Restraint, Very significant enforcement power |
| **Employment Protections** | **LM_IEP W** | Labour Market, Increase of Employment Protection, Weak enforcement power |
|  | **LM_DEP S** | Labour Market, Decrease of Employment Protection, Significant enforcement power |
|  | **LM_DEP V** | Labour Market, Decrease of Employment Protection, Very significant enforcement power |
| **Bargaining Institutions** | **CB_IND W** | Collective Bargaining, Individualised, Weak enforcement power |
|  | **CB_IND S** | Collective Bargaining, Individualised, Significant enforcement power |
|  | **CB_IND V** | Collective Bargaining, Individualised, Very significant enforcement power |
|  | **CB_SOL_W** | Collective Bargaining, Solidaristic, Weak enforcement power |

The coding of the policy orientation of a NEG prescription is based on Table 3 of our article. The coding of the enforcement power of a NEG prescription is based on the country location in the NEG enforcement regime in a given year, as documented in Tables 1 and 2 in the article.
